# Supplementary material for: High-fidelity CRISPR/Cas9- based gene-specific hydroxymethylation rescues gene expression and attenuates renal fibrosis
Source: Nat Commun. 2018 Aug 29;9:3509. doi: 10.1038/s41467-018-05766-5 (PMC6115451; doi:10.1038/s41467-018-05766-5)
Supplement: Supplementary file 1 — Supplementary information [file 41467_2018_5766_MOESM1_ESM.pdf]

High-fidelity CRISPR/Cas9-based gene-specific hydroxymethylation rescues gene expression and attenuates renal fibrosis

Xingbo Xu[1,2,‡], Xiaoying Tan[2,3‡], Björn Tampe[3], Tim Wilhelmi[1,2], Melanie S. Hulshoff[1,2,4], Shoji Saito[3], Tobias Moser[5], Raghu Kalluri[6], Gerd Hasenfuss[1,2], Elisabeth M. Zeisberg[1,2,ɪ,*], and Michael Zeisberg[2,3,ɪ].

-Supplementary Information-

[1]Department of Cardiology and Pneumology, University Medical Center Göttingen, Robert-Koch-Str. 40, 37075 Göttingen, Germany

[2]German Center for Cardiovascular Research (DZHK), Partner Site Göttingen, Germany

[3]Department of Nephrology and Rheumatology, University Medical Center Göttingen, Robert-Koch-Str. 40, 37075 Göttingen, Germany

[4]Department of Pathology and Medical Biology, University Medical Center Groningen, Hanzeplein 1, 9713 GZ Groningen, Netherlands

[5]Institute for Auditory Neuroscience & Inner Ear Lab, University Medical Center Göttingen, Robert-Koch-Str. 40, 37075 Göttingen, Germany

[6]Department of Cancer Biology, Metastasis Research Center, University of Texas, MD Anderson Cancer Center, 1881 East Road, Houston, TX 77054-1901, USA

[‡]Authors contributed equally to this work

[ɪ] share last authorship

Running Title: Cas9-based gene-specific demethylation

Key Words: CRISPR, Cas9, TET3, Rasal1, methylation, hydroxymethylation, fibrosis.

***Correspondence:** Prof. Elisabeth Zeisberg, MD

Department of Cardiology and Pneumology

University Medical Center of Göttingen, Georg-August-University

Robert-Koch-Str. 40

37075 Göttingen, Germany

Telephone: +49-(0)551-3920076

Fax: +49-(0)551-3920077

Email: Elisabeth.zeisberg@med.uni-goettingen.de

Table of Contents

1) Supplementary Figures

Supplementary Figure 1. Expression of mutated Cas9 protein and TET3 catalytic domain

Supplementary Figure 2. Expression of dCas9, dCas9-TET3CD or dCas9-TET3CDi fusion proteins in HEK293 cells

Supplementary Figure 3 Overexpression of TET3 catalytic domain does not restore the expression of 4 different aberrantly methylated genes.

Supplementary Figure 4 Sanger sequencing of bisulfite PCR products of *RASAL1* promoter region in TK188 cells

Supplementary Figure 5 Detection of hydroxymethylation in *RASAL1* promoter induced by dCas9-TET3CD fusion protein by gRES-PCR assay

Supplementary Figure 6 Assessment of the off-target effects of the dCas9-TET3CD-*RASAL1*-sgRNA3 demethylation in TK188 cells

Supplementary Figure 7 Assessment of the off-target effects of the dCas9-TET3CD-*KL*-sgRNA2 demethylation in HK2 cells

Supplementary Figure 8 TGFβ1 induces decreased Rasal1 expression through promoter hypermethylation in mKF

Supplementary Figure 9 Sanger sequencing of bisulfite PCR products of Rasal1 promoter region in TGFβ1-treated mKF

Supplementary Figure 10 Sanger sequencing of bisulfite PCR products of Klotho promoter region in TGFβ1-treated MCT cells

Supplementary Figure 11 Expression of dHFCas9, dHFCas9-TET3CD in HEK293 cells

Supplementary Figure 12 Characterization of *Rasal1*$^{tm1a/tm1a}$ mutant mice

Supplementary Figure 13 Original Western Blots to Main Figure 3c

2) Supplementary Tables

Supplementary Table 1. DNA sequences used for guiding RNA

Supplementary Table 2. Primer sequences used for PCR cloning

Supplementary Table 3. MeDIP primer sequences

Supplementary Table 4. Primer sequences used for *RASAL1* gRES-PCR

Supplementary Table 5. Bisulfite sequencing primer sequences

Supplementary Table 6. Primer sequence for genotyping *Rasal1*^{tm1a} mutant mouse

Supplementary Table 7. qRT-PCR primer sequences

Supplementary Table 8. Gene list of predicted off-targets for human *RASAL1* sgRNA3

Supplementary Table 9. Gene list of predicted off-targets for human *KL* sgRNA2

Supplementary Figure 1

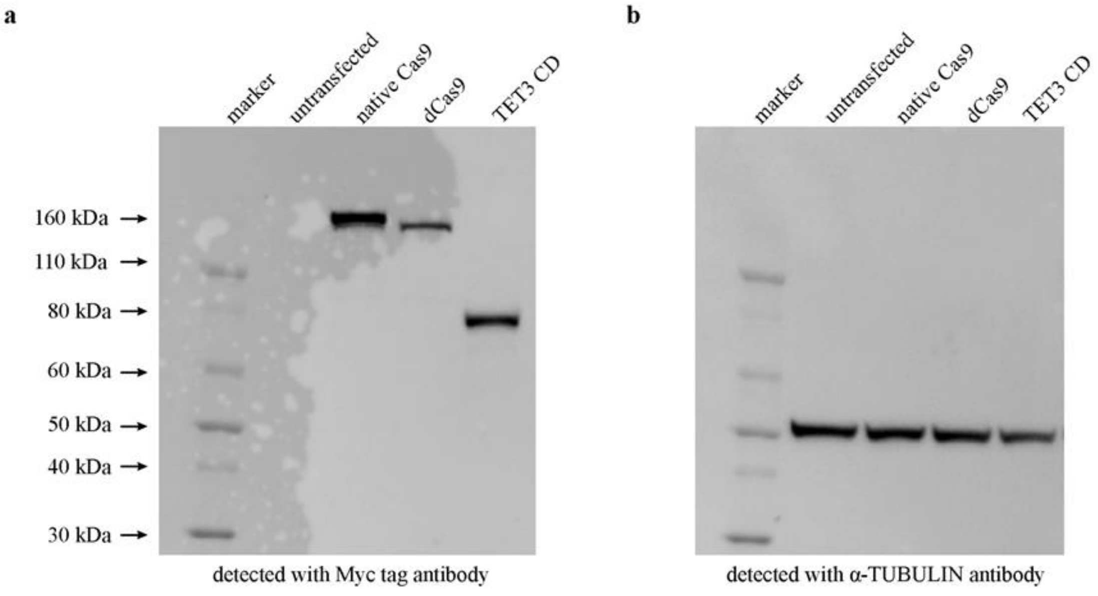

Supplementary Figure 1. Expression of mutated Cas9 protein and TET3 catalytic domain.

(a) Western blot analysis showing the expression of native and mutated Myc-tagged Cas9 (dCas9) protein as well as human TET3 catalytic domain by Myc antibody in HEK293 cells. (b) Untransfected cells served as negative control. The membrane was restripped and re-probed with anti-TUBULIN antibody to serve as loading control.

Supplementary Figure 2

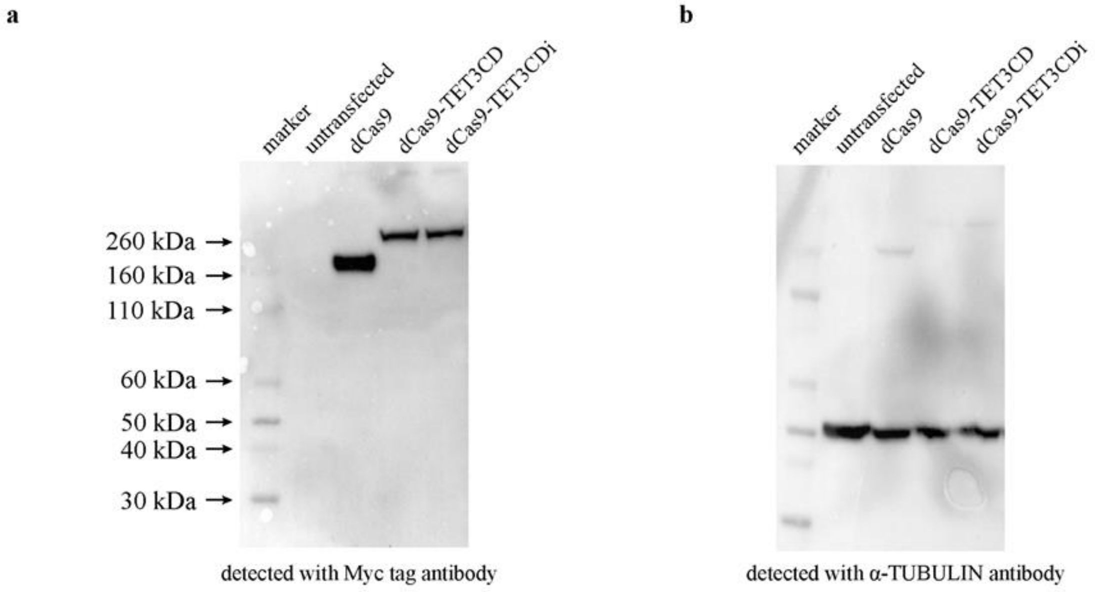

Supplementary Figure 2. Expression of dCas9, dCas9-TET3CD or dCas9-TET3CDi fusion proteins in HEK293 cells.

(a) Western blot analysis showing the expression of dCas9, dCas9-TET3CD or dCas9-TET3CDi fusion proteins by Myc antibody in HEK293 cells. Untransfected cells served as negative control. (b) The membrane was restripped and re-probed with anti-TUBULIN antibody to serve as loading control.

Supplementary Figure 3

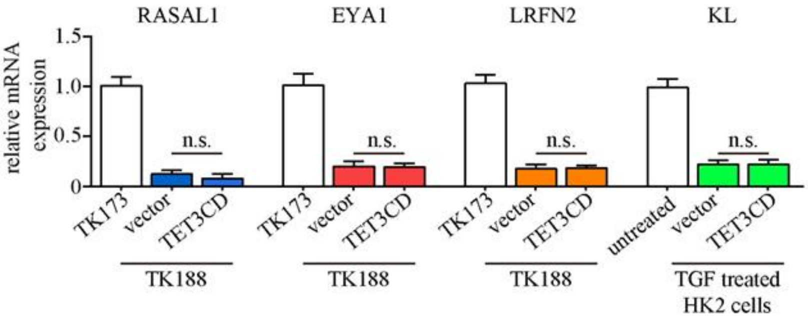

Supplementary Figure 3. Overexpression of TET3 catalytic domain does not restore the expression of 4 different aberrantly methylated genes.

qRT-PCR results showing that only overexpression of TET3 catalytic domain does not induce the expression of 3 different aberrantly methylated genes RASAL1, EYA1, LRFN2 in TK188 fibrotic human kidney fibroblasts and does not induce the expression of KL in TGFβ1-treated HK2 cells. Results were normalized to reference gene GAPDH (expression is presented as mean value; error bars represent S.E.M.; n=3 independent biological replicates).

Supplementary Figure 4

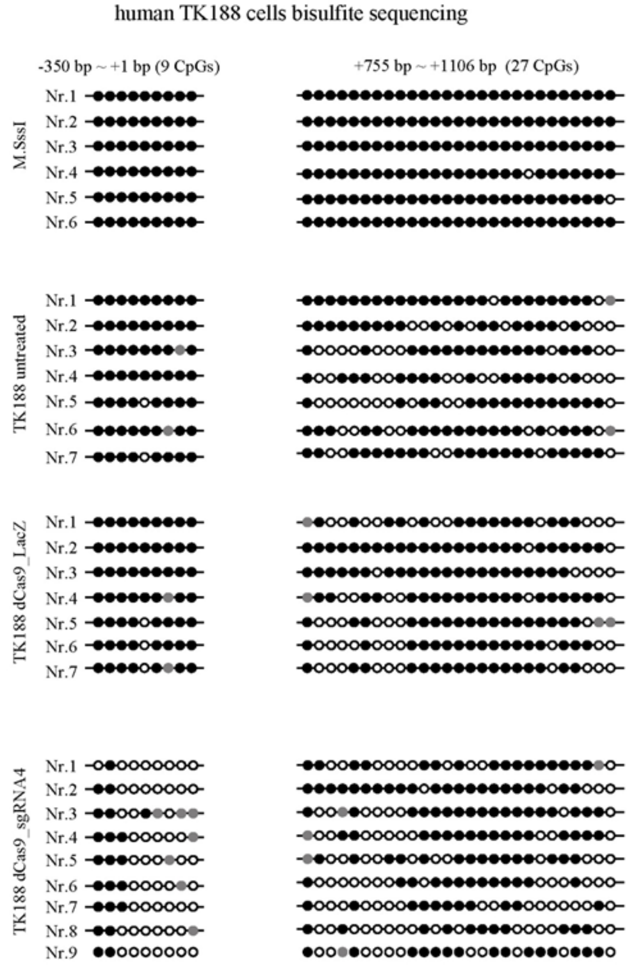

Supplementary Figure 4. Sanger sequencing of bisulfite PCR products of *RASAL1* promoter region in TK188 cells.

Bisulfite sequencing analysis showing the methylation status of CpGs in two different regions (region 1: from -350 bp to +1 bp; region 2: from + 755 bp to +1106 bp) within the *RASAL1* promoter in TK188 cells transduced with lentivirus expressing dCas9-*RASAL1*-sgRNA and control dCas9-*LacZ*-sgRNA constructs. M.SssI treated cell genomic DNA served as bisulfite conversion control. Each panel shows at least six different sequencing results derived from three independent biological replicates. Closed circles indicate methylated, open circles indicate un-methylated and grey circles indicate undetermined CpGs.

Supplementary Figure 5

a

```
1    CAGGAGAGGTGACGCGTGGCCCACGCCTCAAAGGACAACAATCAGAATATCAAACACTTTCTGGACGTGTATTAGGTACTTGTCACTGTC   90
91   CTGAGACTTTTATCTTCATTAACTCTTTTAATTCTTTCAAAATCCTGTGACTGAAATGCAATGTCATCCTCACTCAACAGATGAGAAAAC   180
181  CAAACCAAAGCACAGAGAGGTTGAATGGCTTACCCAAGTCCAGACAGCTACCAAATGGCAGAGCCAGGATTCGAACCCCTGGAAGGCAAA   270
271  TCAGTTACTGACTGTGAGACCTCTGCCAAGTTATTTAATCCCTCTGAACCTCAGTTTCTTTATCCATAAACTGCGGATAATGGGGTAACA   360
361  GTGGAGTAGTGTGTAGTGCACATCTGGAGACAAAAATGAGCTAATCCCAGTAAAGCGCTTAGAACAGTGCCTGGCACCCATGAAGTGTTC   450
451  AAACAACTGTAGATGACTTTTTATCATCGCTGTAGTGGTTATCTTCAAGCCTTCCCCTCCCCCATCTGGCCGCGGCTTCCTCCCCCGCAG   540
541  TGCCAGCCCGTGACGTTAGAGCAGAGTCTGGGTTCTGTGTTTAACTGGAAACTAGAACGAGATGGAAGGGGATGTTCAAGGCCCCTCCCT   630
631  TGACTCTGAACGGACCCCCAGGGAACATGCGACCCTCTCTCTGGCGACGCCTCCCACCCACCACTAATACTTGCTCCTGGACCGGGGGGC   720
721  GCGGAGGTTGGAGAGAGGAGGCAGGTGTCTGCATGGTAGGCAAGGAGGGGTGGGGGCGGAGAGGTGTGAATATGTTGGATGAGGGACAGG   810
811  CGGGGGCACAGACCATGTCTTTTAAGCCCAATGCCAACTCACCAGGAGCCAGCGGCCACGCCCATCAGGACGGGGGCCCCTCCCCACCTG   900
901  GCGGTGCACCCTTGTCTCCGCCGCCCGTGCGCTCTGGCGCGCATGACTGGGGTGCCGGTAGAGGGCGCGCGAGGGGGCCCGGGGTCAGGT   990
991  GGGGGACCCCATTGGAGGCGGGATTGTGGGGAGGTCCCTGGGCCGCCCCTCCCGCCCCCTGCCCGCCCAGCCTGCTTGTCTGGAGCTCCA 1080
1081 GACGCCTCGGCAAGAGCCCGCTGCGCCCCCTGCGCGCTCCTCCTGCTCGGAGCACGGCTCCTACCTGTAGAGTCCGGACGGGCGCTCCCT 1170
1171 CCCCGGGCACCTACACCTTCTCCTCTTTCGGAAATCTCCCATCCAGCTACCCGGGTCTCGGACAGGCGGCACTGGGACCACGAGGCAGGG 1260
1261 AGCCAGGCTTGAAGCAGGTGACATGTAGACGTCCCCTGGTCCAGCCTCGGAACCTGAGCGCCCTTCTGCCTGGAAAGTTTGTGGCTAGGC 1350
1351 GCCATGGCCAAGAGCAGCTCCCTGAATGTTCGCGTGGTGGAGGGCCGCGCGCTGCCTGCCAAGGACGTGTGAGTACTCCTCGGGCGCTTC 1440
1441 TGGGTGAGGGGCGCGCAAGGCCTGGGGTGGGGCCGACGTCGCCCTTTCCGCGTGCCAGGGCGGGTTGGCAGGAGGGCGAGGGGTTCTTGT 1530
1531 CCAAGACTGGTCTTGGGGCAGTTCCGCTTTAAAGGGGCGAGAGGCCGCATGCCCATGGCTGTCCCGGTGGAGGGGGGCGCCTAGAGTTA 1620
1621 GCAGATGCCCCGCCTCACCCCCGGGCAGGGGAGGGGGTGTCTTTGTTCCCGGGATGTACTAAAGGGGGTCGTCCAGAGCTGAAGAGGGGA 1710
1711 TTAGAGGGCCATATTCTGTCAGATCTGGGAGGGGAGAAAGCGGGGGGTCTCTTCCTTAGTCCAGGCAGATGTTGGAGGTGGCTCCTCCAG 1800
```

MspI/HapII cutting site: CCGG

Exons

Start codon: ATG

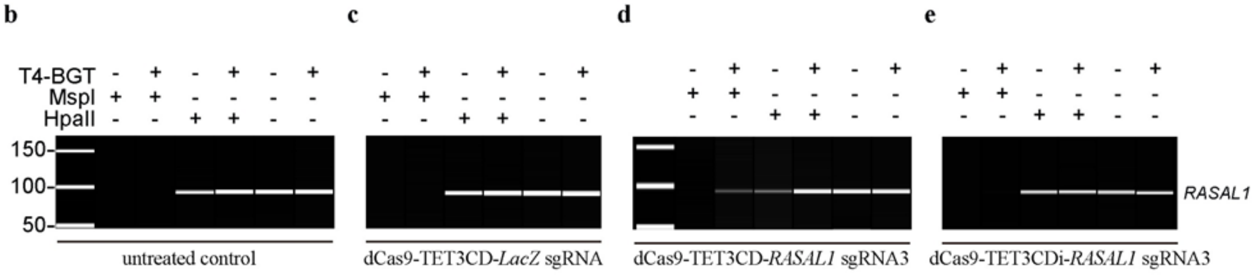

Supplementary Figure 5. Detection of hydroxymethylation in *RASAL1* promoter induced by dCas9-TET3CD fusion protein by gRES-PCR assay.

(a) DNA sequence of the *RASAL1* promoter region with exons highlighted in yellow, MspI/ HapII restriction sites in blue, CG dinucleotide highlighted in bold and the start codon in red. The primers used for the glucosylation-mediated restriction enzyme sensitive PCR (gRES-PCR) are framed. (**b-e**) Hydroxymethylation is detected by glucosylation-mediated restriction enzyme sensitive PCR (gRES-PCR) assay in TK188 cells transduced by lentivirus expressing dCas9-TET3CD-*LacZ*/*RASAL1*-sgRNA or dCas9-TET3CDi-*RASAL1*-sgRNA demethylation constructs. DNA virtual gel images present the *RASAL1* PCR products from different combinations of enzymatic treatment. T4-BGT transfers glucose specifically to hydroxymethylated DNA, MspI cleaves hydroxymethylated DNA without but not with glucose and HpaII cleaves only native DNA. Only cells transduced with the dCas9-TET3CD-*RASAL1*-sgRNA3 construct show a *RASAL1* PCR band after treatment with T4-BGT/MspI, but not in dCas9-TET3CD-*LacZ*-sgRNA nor in dCas9-TET3CDi-*RASAL1*-sgRNA3 transduced cells, confirming specific hydroxymethylation of *RASAL1* is dependent on *RASAL1*-sgRNA3 and TET3CD activity.

Supplementary Figure 6

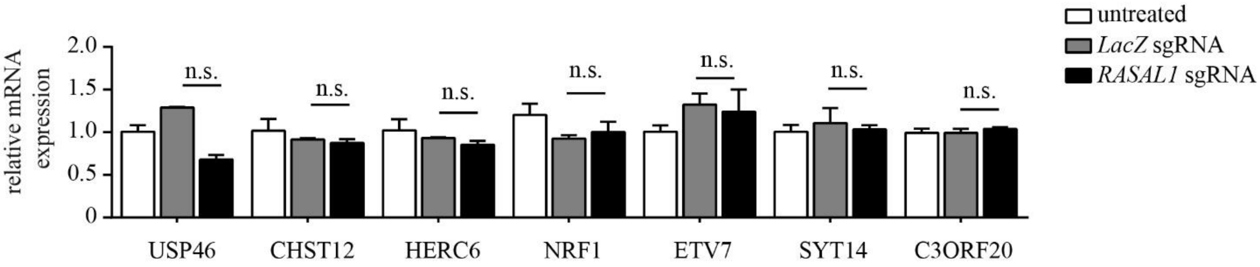

Supplementary Figure 6. Assessment of the off-target effects of the dCas9-TET3CD-*RASAL1*-sgRNA3 demethylation in TK188 cells.

Human fibrotic TK188 cells were transduced with lentivirus expressing demethylation constructs guided by *RASAL1*-sgRNA3 or by *LacZ*-sgRNA. The mRNA expression of predicted off-target genes was assessed by qRT-PCR analysis, but there is a not significant difference between *RASAL1*-sgRNA3 and *LacZ*-sgRNA transduced cells for all tested genes. Results were normalized to reference gene GAPDH (expression is presented as mean value; error bars represent S.D.; n = 3 independent biological replicates; n.s., not significant).

Supplementary Figure 7

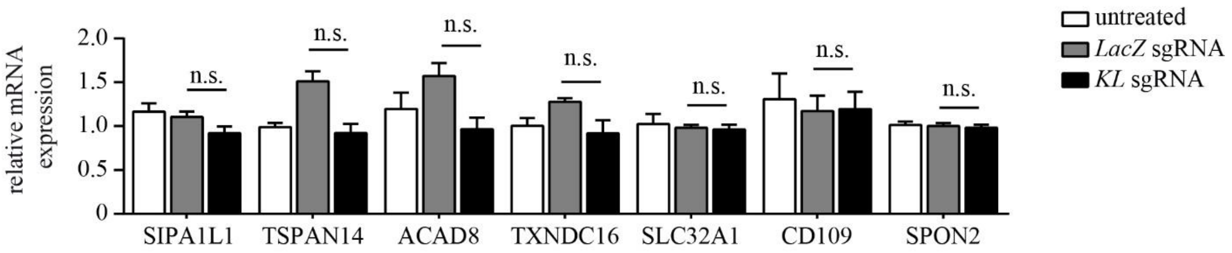

Supplementary Figure 7. Assessment of the off-target effects of the dCas9-TET3CD-*KL*-sgRNA2 demethylation in HK2 cells.

TGFβ1-treated HK2 cells were transduced with lentivirus expressing demethylation constructs guided by *KL*-sgRNA2 or by *LacZ*-sgRNA. The mRNA expression of predicted off-target genes was assessed by qRT-PCR analysis, but there is no significant difference in gene expression between *KL*-sgRNA2 and *LacZ*-sgRNA transduced cells for all tested genes. Results were normalized to reference gene GAPDH (expression is presented as mean value; error bars represent S.D.; n = 3 independent biological replicates; n.s., not significant).

Supplementary Figure 8

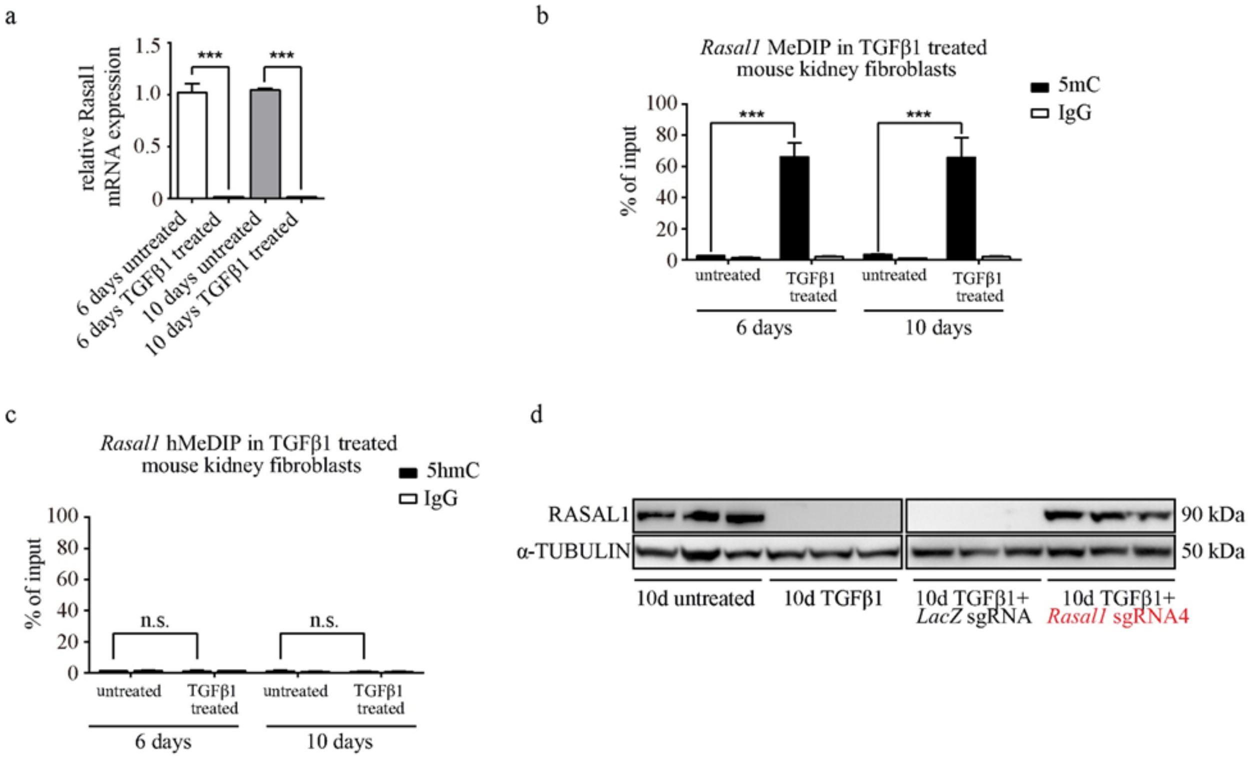

Supplementary Figure 8. TGFβ1 induces decreased Rasal1 expression through promoter hypermethylation in mKF.

(**a**) Mouse kidney fibroblasts (mKF) were treated with TGFβ1 for 6 and 10 days. The Rasal1 mRNA expression was analysed by qRT-PCR assay. Results were normalized to reference gene Gapdh (expression is presented as mean value; error bars represent S.D.; n = 3 independent biological replicates; ***, $p < 0.001$). (**b**) MeDIP-qPCR analysis showing a significant increase of *Rasal1* promoter methylation in TGFβ1-treated mKF compared to non-treated control mKF. The results were calculated relative to input. The data is presented as mean value; error bars represent S.D.; n=3 independent biological replicates; ***, $p < 0.001$. (**c**) hMeDIP-qPCR analysis shows no significant change in *Rasal1* promoter hydroxymethylation levels in TGFβ1-treated mKF in comparison to non-treated control mKF. (**d**) Western blot analysis shows the restored RASAL1 protein expression in TGFβ1-treated mKF which are transduced with lentivirus expressing *Rasal1*-sgRNA but not with control *LacZ*-sgRNA.

Supplementary Figure 9

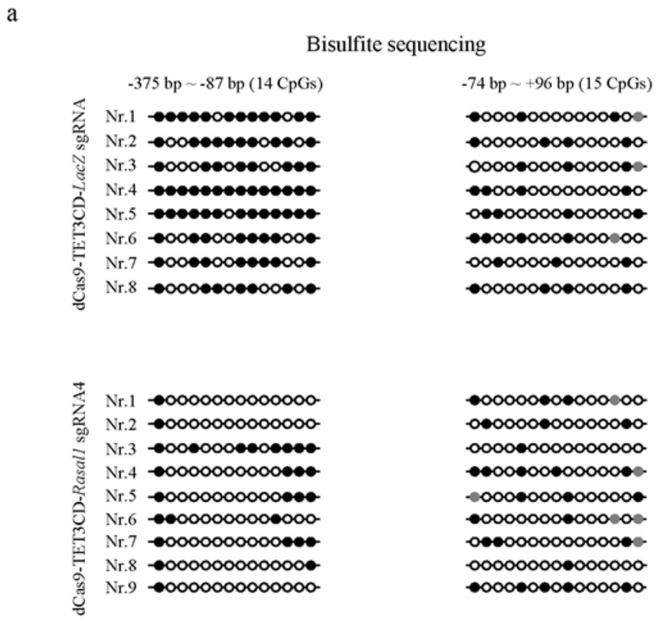

Supplementary Figure 9. Sanger sequencing of bisulfite PCR products of *Rasal1* promoter region in TGFβ1-treated mKF.

Bisulfite sequencing analysis showing the methylation status of CpGs in two different regions (region 1: from -375 bp to -87 bp; region 2: from -74 bp to +96 bp) within the *Rasal1* promoter in TGFβ1-treated mKF transduced with lentivirus expressing dCas9-*Rasal1*-sgRNA4 and control dCas9-*LacZ*-sgRNA constructs. Each panel shows at least six different sequencing results derived from three independent biological replicates. Closed circles indicate methylated, open circles un-methylated and grey circles undetermined CpGs.

Supplementary Figure 10

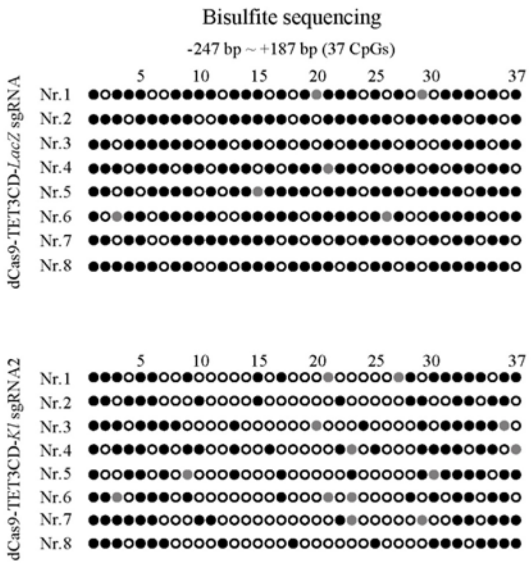

Supplementary Figure 10. Sanger sequencing of bisulfite PCR products of *Klotho* promoter region in TGFβ1-treated MCT cells.

Bisulfite sequencing analysis showing the methylation status of CpGs in *Klotho* promoter regions from -247 bp to +187 bp) in TGFβ1-treated renal tubular epithelial cell (MCT) cells transduced with lentivirus expressing pLenti-dCas9-*Klotho*-sgRNA2 and control pLenti-dCas9-*LacZ*-sgRNA constructs. Each panel shows at least six different sequencing results derived from three independent biological replicates. Closed circles indicate methylated, open circles un-methylated and grey circles undetermined CpGs.

Supplementary Figure 11

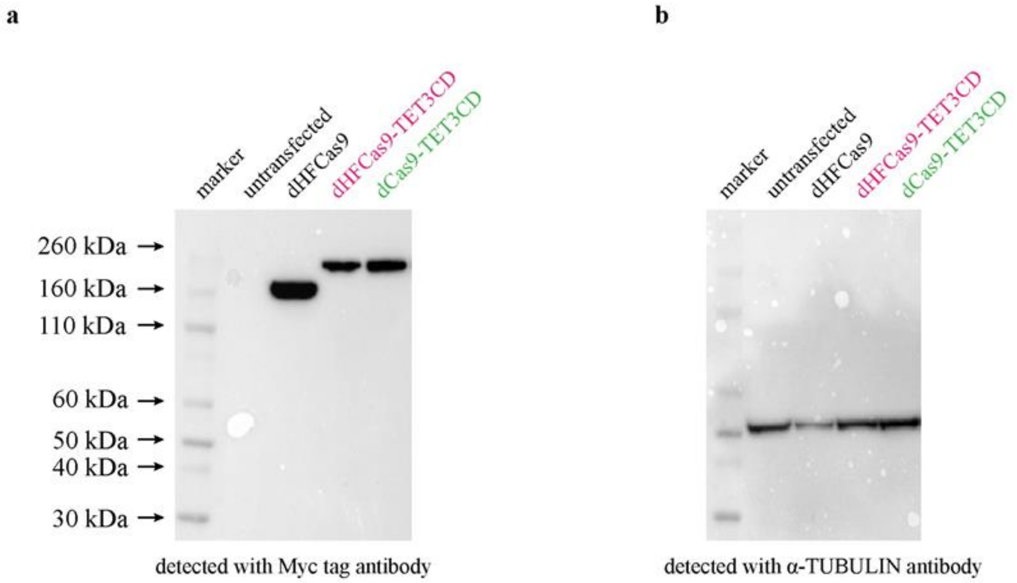

Supplementary Figure 11. Expression of dHFCas9, dHFCas9-TET3CD in HEK293 cells.

(a) HEK293 cells were transfected with dHFCas9, dHFCas9-TET3CD or dCas9-TET3CD constructs and detected with Myc antibody. Untransfected cells served as negative control. (b) The membrane was restripped and re-probed with anti-TUBULIN antibody to serve as loading control.

Supplementary Figure 12

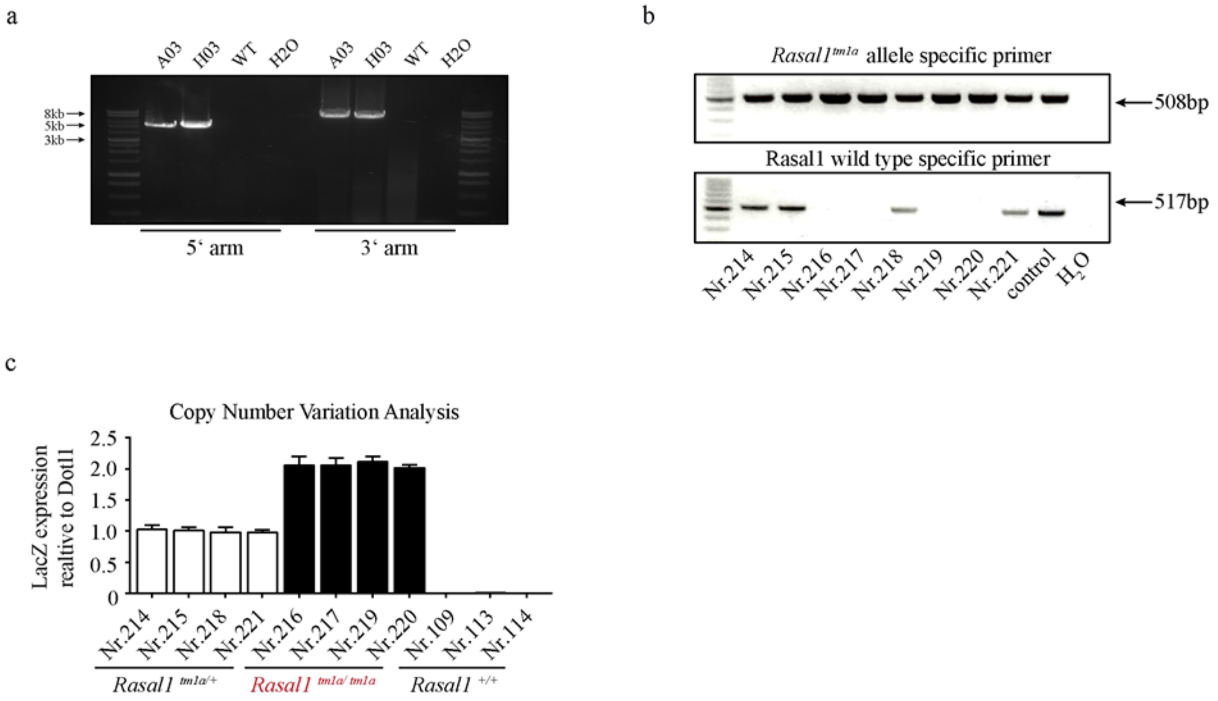

Supplementary Figure 12. Characterization of *Rasal1*$^{tm1a/tm1a}$ mutant mice

(**a**) Agarose gel picture shows the expected size of long-range PCR products from *Rasal1*^{tm1a} mutant ES cells (2 different clones A03, H03) tested from both 5' and 3' directions. (**b**) Agarose gel picture shows the genotyping PCR products from genomic DNA of *Rasal1* ^{tm1a} homozygous and heterozygous mice. (**c**) qRT-PCR analysis shows the copy number variation in *Rasal1* ^{tm1a} mice assayed with a *LacZ* probe. Results were normalized to reference gene Dotl1 (expression is presented as mean value; error bars represent S.D.).

Supplementary Figure 13

a

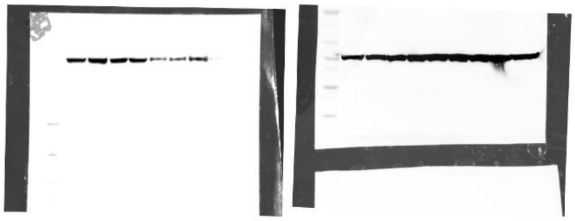

b

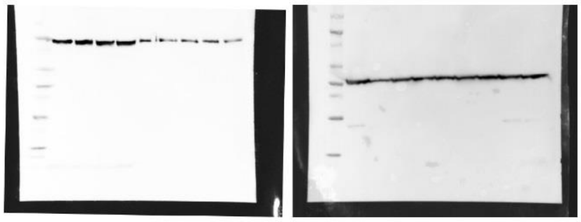

c

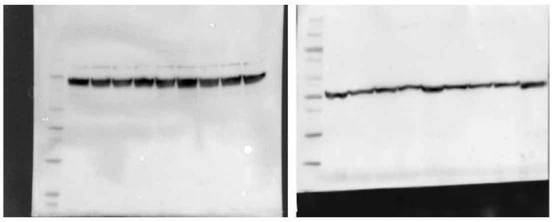

d

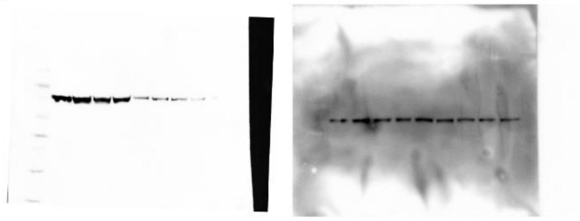

e

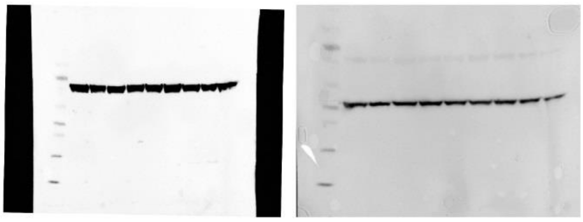

f

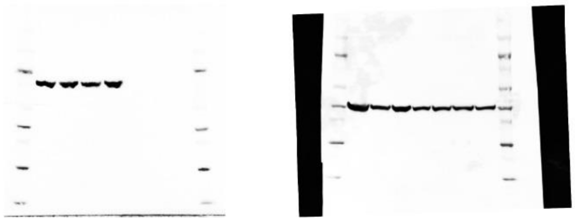

g

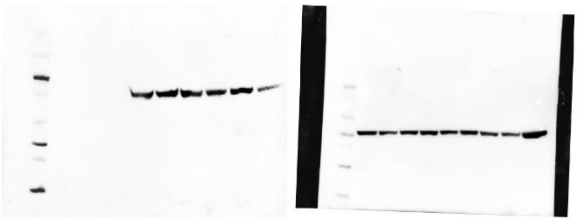

Supplementary Figure 13. Uncropped Western blots. (a) Original Western blots for Main Figure 3c, RASAL1 protein (left) and α-TUBULIN protein (right). (b-e) Original Western blots for Main Figure 5d, RASAL1 protein (left) and α-TUBULIN protein (right). (f-g) Original Western blots for Supplementary Figure 8d, RASAL1 protein (left) and α-TUBULIN protein (right).

Supplementary Table 1: DNA sequences used for guiding RNA

**Human RASAL1**	
sgRNA1	CAAAGCACAGAGAGGTT*GAA*
sgRNA2	ATCCATAAACTGCGGATAAT*GGG*
sgRNA3	ACTTTTTATCATCGCTGTAG*TGG*
sgRNA4	GGAACATGCGACCCTCTCTC*TGG*
sgRNA5	AAGCCCAATGCCAACTCACC*AGG*
sgRNA6	ACTGTTCTAAGCGCTTTACT*GGG*
sgRNA7	CACTGCGGGGGAGGAAGCCG*CGG*
sgRNA8	GGTCCAGGAGCAAGTATTAG*TGG*
sgRNA9	GGCATTGGGCTTAAAAGACA*TGG*
sgRNA10	AGTACTCACACGTCCTTGGC*AGG*

**Human EYA1**	
sgRNA1	AGCTGTTTATTCAGATAGCG*TGG*
sgRNA2	TTAGGACAAGAGAATAGCTG*TGG*
sgRNA3	TCTGCCGCTGCTGTTTGGTG*GGG*
sgRNA4	AGAATAACTTCTCTGAGACG*TGG*
sgRNA5	ATTTGTTTGCGCAGCCTTGT*AGG*
sgRNA6	TCAGCTCCCACCGTTCTGTT*TGG*

**Human LRFN2**	
sgRNA1	CCCACCTCCCTCCGCGACTG*CGG*
sgRNA2	CTTCGGCTCCTGCAGCTCCG*CGG*
sgRNA3	CGCCCGACTCCCTCCCAGAT*TGG*
sgRNA4	ATGCCTGGGGTTCTTCGATC*CGG*
sgRNA5	AACACCGCCGCGGAAACCAG*AGG*
sgRNA6	GCCAGAGGCGCCCAGGAACC*CGG*
sgRNA7	TCAAGTTCAGACGAGCCTGC*CGG*
sgRNA8	TGGCATTTAGCAAGAAGAAG*GGG*

**Human KL**	
sgRNA1	CTCCCCGACGAAGCCGCTCC*AGG*
sgRNA2	TCGCAGGTAATTATTGCCAG*CGG*
sgRNA3	TTCCAGGGCACCTTCCCCGA*CGG*
sgRNA4	TACGTTCACCCACCACCCCC*TGG*
sgRNA5	CGCTCCCCGGCGGGCTCCGC*TGG*
sgRNA6	ACCAGCAGCAGCGACAGCGA*CGG*
sgRNA7	CCAGAGGAAGCCGTCGGGGA*AGG*
sgRNA8	GACGTTGTTGTAGCTGTCGC*TGG*

**Mouse Rasal1**	
sgRNA1	CATGACATACAGAGTTGCCG*AGG*
sgRNA2	CGCAGTGGCAGCCGGTGACG*TGG*
sgRNA3	GCCCAGAGAGAGTACTGTTC*CGG*
sgRNA4	GAGACACCCGGGTTCTTGGA*GGG*
sgRNA5	CGAACACTTAACCAAGTTCC*AGG*
sgRNA6	TTAAACCCGGAGTGTGCCAG*CGG*
sgRNA7	CAACACCAGCCTCGGGGTCC*CGG*
sgRNA8	GAGCAGTCAAGCAGGGCTGC*AGG*

**Mouse Kl**	
sgRNA1	CTCCCTGAGCTGGCTGCAGC*AGG*
sgRNA2	TGTGCTCTCTCTGGGCACCC*TGG*

sgRNA3	CCCCCGTGCCAGGCCGGAGT*GGG*
sgRNA4	AGCGGGGGTGGGCACCGCGT*AGG*
sgRNA5	CGCTGCCTGAGCGCTGAGCC*GGG*
sgRNA6	GCTCCTGCCCCAGAGGCCGC*TGG*

Supplementary Table 2: Primer sequences used for PCR cloning

**Cas9 cloning primer**	
NlsmutCas9_F:	TTAAACCGGTGAGGAGATCTGCCGCCGCGATCGCCATGGCGCCG AAAAAAAAACGCAAAGTGGGCCGCGGCATGGATAAGAAATACT CAATAGGCTTAGCTATCGGCACAAATAGCGTCGGAT
NlsmutCas9_R:	TTAAGCGGCCGCGTTCTAGACACTTTGCGTTTTTTTTTCGGGTCA CCTCCTAGCTGACTCAAATC
**TET3 catalytic domain cloning primer**	
hTet3CDAge1Xba1_F:	TTAAACCGGTATGTCTAGAGGCGGAGGCTCGGGCTATTATACTCA CTTGGGATCTGGCCCCA
hTET3CD_R1:	TTAAGCGGCCGCGTAGCCCCAAACAGCTTCTCGCTGGAT
**TET3 catalytic domain mutation primers**	
hTET3CDTM_F	TGTGCCCACGCCTACAAGGCCCAGCATAAC
hTET3CDTM_R	GAAGTCCATGCAGGCCGTGACCC

Supplementary Table 3: MeDIP/hMeDIP primer sequences

Mouse MeDIP/hMeDIP primer		
**Name**	**Sequence**	**Reference**
mRasal1_MeDIP_F1:	TGCAGATGGCTCTTATCGTG	
mRasal1_MeDIP_R1:	CCAATTAAACCCGGAGTGTG	
MeDIP_mKL_F1:	AAACCTCGCAAAGTTCCACC	
MeDIP_mKL_R1:	CAGAAACAGCTGCCCAACTT	
**Human MeDIP/hMeDIP primer**		
**Name**	**Sequence**	**Reference**
hRasal1_MeDIP_F1:	GCCAACTCACCAGGAGCCAGCGGC	Tampe et al., *EBiomedicine*, 2015
hRasal1_MeDIP_R1:	CTACCGGCACCCCAGTCATGCGC	
hKL_MeDIPF1:	CCAGTCCCTAATTGGCTCCA	
hKL_MeDIPR1:	GACCAACTTTCCCCGACTTG	

Supplementary Table 4: Primer sequences used for RASAL1 gRES-PCR

RASAL1 gRES-PCR primers	
hRasal1_EpiMrkF1:	ACTGGGGTGCCGGTAGAG
hRasal1_EpiMrkR1:	AGGGACCTCCCCACAATC

Supplementary Table 5: Bisulfite sequencing primer sequences

**Mouse bisulfite sequencing primers**		
**Name**	**Sequence**	**Reference**
mouse Rasal1 bisu F4:	ATTGGAGACGAAGAGAAGATGTAAGGGGATG	
mouse Rasal1 bisu R4:	AACGCCCCCTACTAACACCCCAAACTACTAC	
mouse Rasal1 bisu F9:	GGGGGTTTTCGGTTGAGTGTAGTAGTTTGGG	
mouse Rasal1 bisu R9:	AACATACGTCCAAAACTACAAATAACTATAC	
BismKLF3:	GGATTGTGTGATGTGGAATAGTTTGTTTTTTGAG	
BismKLR3:	AACAACAACAAATACAACAACAACAAAC	
BismKLF6:	TTTTAGGAATATTAGTTTTAGGAAGGTAAAGGG	
BismKLR6:	CCCACGAACAATAATTATCCAAAACAAAC	
**Human bisulfite sequencing primers**		
**Name**	**Sequence**	**Reference**
human RASAL1 BGS2 F:	TAGTTTGTTTGTTTGGAGTTTTAGA	Bechtel et al., *Nat Med*, 2010
human RASAL1 BGS2 R:	ACCTACTTCAAACCTAACTCCCTAC	
human RASAL1 bisu F2:	AGTTAGGATTCGAATTTTTGGAAGGTAAATTAG	
human RASAL1 bisu R2:	TTTCCAATTAAACACAAAACCCAAACTCTACTC	
human RASAL1 bisu F5:	GCGTTATGGTTAAGAGTAGTTTTTTGAATG	
human RASAL1 bisu R5:	CTCTAAACAACCCCCTTTAATACATCCC	

Supplementary Table 6: Primer sequence for genotyping Rasal1^{tm1a} mutant mouse

Rasal1^{tm1a} mutant mouse genotyping primers		
**Name**	**Sequence**	**Reference**
Rasal1-F	TGTCTGGCTTTGACTTGACCCTAGC	IMPC
Neo-F	GGGATCTCATGCTGGAGTTCTTCG	
Rasal1-ttR	ACACATCCAGACATGCAAAGGAAGC	
LacZ assay-F	CTCGCCACTTCAACATCAAC	IMPC
LacZ assay-R	TATCAGCCGGAAAACCTACC	
LacZ assay-Probe	TCGCCATTTGACCACTACCATCAATCC	
Dot1l control-F	GCCCCAGCACGACCATT	IMPC
Dot1l control-R	TAGTTGGCATCCTTATGCTTCATC	
Dot1l control-Probe	CCAGCTCTCAAGTCG	
CSD-RAF5-F	CACACCTCCCCCTGAACCTGAAAC	IMPC
GF2	GTCTTACAGGACGGGTTAAAGGAG	
LR-5En2frt-R	GGTGGTGTGGGAAAGGGTTCGAAG	
GR1	GAGCCAAGGGTCCAATGAGATGAC	

Supplementary Table 7: qRT-PCR primer sequences

**Primers for mouse genes**		
**Name**	**Sequence**	**Reference**
qRT-mRasal1_F:	CTCACAAGGCGTGAGGTGG	Bechtel et al., *Nat Med*, 2010
qRT-mRasal1_R:	TGCCAAGGAGTTAGAACGGAA	
qRT-mKl_F:	ACTACGTTCAAGTGGACACTACT	Primer bank
qRT-mKl_R:	GATGGCAGAGAAATCAACACAGT	
**Primers for human genes**		
**Name**	**Sequence**	**Reference**
qRT_hUSP46_F:	TCCGGGAGAATGTGTTGGC	Primer bank
qRT_ hUSP46_R	GTGTGGCAATGCTGTGGAAAA	
qRT_hCHST12_F:	CTTCTACTTGCACACGTCCTT	Primer bank
qRT_hCHST12_R:	CTCCGTCTCCTTTCTGGGAA	
qRT_hHERC6_F:	CCACTCCCTGGCATTATCAAAA	Primer bank
qRT_hHERC6_R	GCCAAACGAAGTCCCACAGA	
qRT_hNRF1_F:	AGGAACACGGAGTGACCCAA	Primer bank
qRT_hNRF1_R:	TATGCTCGGTGTAAGTAGCCA	
qRT_hETV7_F:	CTGCTGTGGGATTACGTGTATC	Primer bank
qRT_hETV7_R:	GTTCTTGTGATTTCCCCAGAGTC	
qRT_hSYT14_F:	AAATACAGTCCTCTATCGGCAGA	Primer bank
qRT_hSYT14_R:	TTGGGCACTTGTTATATGAGCAT	
qRT-hC3ORF20_F:	GGAAAGAGTCCCTCGCAAACA	Primer bank
qRT-hC3ORF20_R:	GGGGTTCCCACTCCTCGAT	
qRT_mSIPA1L1_F:	GTCGCAGACCGAAAGACCTG	Primer bank
qRT_mSIPA1L1_R:	GGTTGAGGTGATATGGTGAGGG	
qRT_mTSPAN14_F:	GGCTGGCTGGAGTTGTCTTC	Primer bank
qRT_mTSPAN14_R:	GGTCGATTCCATGCAACCG	
qRT_mACAD8_F:	TCCTTGGGGCTAAATGAAGAAC	Primer bank
qRT_mACAD8_R:	TCCCGAGCTGCAAAGTCAAAG	
qRT_mTXNDC16_F:	AGAAGCGTCAAGGTACTGTGG	
qRT_mTXNDC16_R:	GAAGGTCTTCCAGGGTGGTAA	
qRT_mSLC32A1_F:	ACCTCCGTGTCCAACAAGTC	Primer bank
qRT_mSLC32A1_R:	CAAAGTCGAGATCGTCGCAGT	
qRT_mCD109_F:	TCCCGCTTTCTGGTGACAG	Primer bank
qRT_mCD109_R:	ACCTGAGCCTTTACAAGGACC	
qRT_mSPON2_F:	ATGGAAAACGTGAGTCTTGCC	Primer bank
qRT_mSPON2_R:	TGATGCTGTATCTAGCCAGAGG	

Supplementary Table 8: Gene list of predicted off-targets for human *RASAL1* sgRNA3

Coordinates	strand	Mismatches	target_seq	PAM	distance		gene name
chr12:113574133-113574155	-	0	ACTTTTTATCATCGCTGTAG	TGG	89	-	RASAL1
chr20:57696895-57696917	-	3	AGTATTTATCATTGCTGTAG	AGG	8209	-	MRPS16P2
chr4:53515136-53515158	-	4	TCTTCTAATCATTGCTGTAG	TGG	1980	I	USP46
chr7:2467215-2467237	-	4	TTTTTCTATCATCTCTGTAG	GGG	4961	I	CHST12
chr11:88418860-88418882	-	3	ACTTTTAGTCATGGCTGTAG	TGG	32289	I	GRM5
chr9:3046135-3046157	+	4	TCATTTTATCTTAGCTGTAG	AGG	7130	I	CARM1P1
chr2:123688625-123688647	+	4	TATTTTTATAATCTCTGTAG	GGG	NA	-	NA
chr4:89329458-89329480	-	4	ACAATTTATTATCTCTGTAG	TGG	196	I	HERC6
chr7:129298490-129298512	-	4	AATTTGTATTATCCCTGTAG	AGG	1076	I	NRF1
chr13:85978922-85978944	+	4	GCTATTTATCACCCCTGTAG	AGG	40692	I	LINC00351
chr17:70613720-70613742	+	4	AGTTTTTCTCGTCTCTGTAG	AGG	713	I	LINC00511
chr6:36343417-36343439	-	4	ACTTGTTTTCAGAGCTGTAG	GGG	209	I	ETV7
chr1:29280765-29280787	+	4	GCTATTTATCATTGTTGTAG	AGG	28003	I	RP4-604A21.1
chr1:210327126-210327148	-	4	ACTTTTAGTCAACACTGTAG	TGG	1918	I	SYT14
chr8:105743730-105743752	+	4	ATCTTTTATCATCATTGTAG	CGG	16527	I	RP11-127H5.1
chr13:106601077-106601099	-	4	ACTTCAGATCATCGCTGAAG	AGG	51085	-	SNORA25
chr3:14725729-14725751	+	4	GCTTTCTATCATTGCTTTAG	TGG	0	E	C3orf20
chr11:4224812-4224834	+	4	CCTTTTCATCTTCGCTGGAG	CGG	927	-	RP11-23F23.2
chr17:69852904-69852926	+	4	TCTTTATATCATCTCTTTAG	GGG	NA	-	NA
chr13:54376908-54376930	+	4	ACAATTTATCATGGCTGGAG	AGG	12624	-	LINC00558

Potential off-target sites predicted for *RASAL1*-sgRNA3 by the online program CCTop were listed according to the number of mismatched nucleotides. The genome location and the nearby genes are included in the list (E: exonic; I: intronic; -: intergenic).

Supplementary Table 9: Gene list of predicted off-targets for human *KL* sgRNA2

Coordinates	strand	Mismatches	target_seq	PAM	distance		gene name
chr13:33590446-33590468	+	0	TCGCAGGTAATTATTGCCAG	CGG	0	E	KL
chr14:71977318-71977340	+	4	TGCCTGGGAATTATTGCCAG	GGG	2120	I	SIPA1L1
chr5:102757803-102757825	-	4	GCTCAGTTGATTATTGCCAG	TGG	8282	-	AC010423.1
chr20:50965499-50965521	+	4	TTGCAAGATATTATTGCCAG	AGG	6340	I	RP4-723E3.1
chr3:2481671-2481693	-	4	TCTCATTTACTTATTGCCAG	AGG	71341	I	CNTN4
chr6:46878083-46878105	+	4	TTGTGGGTAATAATTGCCAG	TGG	1268	I	RP3-365O12.2
chr10:82250548-82250570	+	4	TAGCATGTGTTTATTGCCAG	TGG	1478	I	TSPAN14
chr11:27423007-27423029	-	4	TTGCATGTGTTTATTGCCAG	AGG	8942	I	LGR4
chrX:134831527-134831549	-	4	TGGCAGTCAAGTATTGCCAG	AGG	11661	-	RP11-432N13.4
chrX:134783498-134783520	-	4	TGGCAGTCAAGTATTGCCAG	AGG	23786	-	RP11-432N13.4
chr4:132263571-132263593	-	4	TAGCTGTTAATTTTTGCCAG	TGG	37575	-	RP11-314N14.1
chr11:134132511-134132533	+	4	TAGAAGGTAAAAATTGCCAG	AGG	0	E	ACAD8
chr14:52901283-52901305	+	4	TCTCAAGTAAGAATTGCCAG	TGG	1978	I	TXNDC16
chr8:54810698-54810720	-	4	AGGCAGGGAATTATAGCCAG	GGG	10894	I	RP11-1070A24.1
chr20:37350290-37350312	+	4	TGGCATCTAATTATAGCCAG	CGG	2793	-	SLC32A1
chr10:84162887-84162909	+	4	TGCTAGGTAATTATTTCCAG	AGG	44263	I	NRG3
chr11:8234267-8234289	+	4	TCAAAGCTAATTATTACCAG	AGG	11562	-	LMO1
chr6:74411096-74411118	+	4	CAGCAGGTAATAATTACCAG	AGG	3801	I	CD109
chrX:65593743-65593765	-	4	TCACAGCTAAATATTACCAG	AGG	12125	-	RP13-238N7.2
chr4:1193268-1193290	-	4	TTCCAGGTGATTATTGCTAG	AGG	0	E	SPON2

Potential off-target sites predicted for *KL*-sgRNA2 by the online program CCTop were listed ccording to the number of mismatched nucleotides. The genome location and the nearby genes are included in the list (E: exonic; I: intronic; -: intergenic).
